# Emerging role of robotics in urology

**DOI:** 10.4103/0972-9941.19268

**Published:** 2005-10

**Authors:** Rajeev Kumar, Ashok K. Hemal

**Affiliations:** Department of Urology, All India Institute of Medical Sciences, New Delhi, India

**Keywords:** Robotic surgery, robot, da Vinci^®^, radical prostatectomy, laparoscopy

## Abstract

Robotic assistance is one of the latest additions to the field of laparoscopic surgery. The most commonly used robotic device in Urology is the da Vinci^®^ system of which over 200 devices are installed worldwide including 3 in India. This robot consists of three or four arms, one of which is used to hold and manipulate the laparoscopic camera while the others are used to manipulate specialized laparoscopic instruments with endowrist^®^ technology that allows 7 degrees of freedom.

The robot is currently used primarily for radical prostatectomies where complex dissection and reconstruction can be performed in less than 2 hours with excellent outcomes. There is a progressive increase in the number of surgeries being performed by this device which allows laparoscopy naïve surgeons to offer the benefits of minimally invasive surgery to their patients. The other surgeries where this device has been used to benefit are pyeloplasty, cystectomy with urinary diversion, nephrectomy and ureteric re-implant.

The principal drawbacks of the device are the steep cost of machine and disposables. However, the benefits achieved in terms of improved surgical precision, magnified 3 dimensional vision, scaling of movements, remote surgery and as a teaching tools will help the robot establish a definitive place in the urologic armamentarium.

## INTRODUCTION

Necessity has always been the mother of invention. This is amply highlighted by the developments in the field of laparoscopy and the rapid improvements in the instrumentation for these surgeries. Limitations of traditional laparoscopy such as two-dimensional vision, limited movement of instruments, complex reconstruction and difficult suturing and also surgeon fatigue have prompted the introduction and rapid assimilation of robotic systems in laparoscopic urology.

A robot is defined as a computerized system with a motorized part, usually an arm that is capable of interacting with the environment. Depending on the amount of surgeon-machine interaction, robotic surgery can be further divided into shared-control, telesurgical and supervisory-controlled. In the shared-control system, the robot simply offers steady-hand manipulations of an instrument placed in its arms. The surgeon directly carries out the procedure and the robot, more or less, replaces an assistant. In a telesurgical system, the surgeon does not directly operate but manipulates the robotic arms during the procedure and the robot is like an instrument in his hands. Using real-time image feedback, the surgeon may operate from a remote location using sensor data from the robot. Because technically the robot is performing the procedure, it is considered robotic surgery. In a supervisory-controlled system, the surgeon feeds directions into the computer before the procedure and the robot then carries out the instructions without any surgeon interference during the actual procedure. Since the robot performs the entire procedure based on a predetermined set of commands, the programming has to be carefully done individually before each surgery.

### History of robotics[1]

Computer Motion Inc., a high-tech medical devices company was founded in 1989 in order to develop newer surgical systems and devices that could improve surgical practices. The first robot-assisted human hip replacements were performed in California using Robodoc- a device manufactured by Integrated Surgical Systems in 1992. The first commercially used robotic assistant, the Aesop™ 1000, was developed in 1993 by Computer Motion. This device worked on the shared-control principle and was used for holding an endoscopic camera in minimal invasive laparoscopic surgery. The device was controlled by foot pedals that were often a problem for new users as they had to look down on the pedals before they could adjust them. The device was modified in 1996 and the Aesop 2000 used voice control while the Aesop 3000 added another degree of freedom in the arm. The Aesop HR version was networked with other smart devices. These devices are still in use at some centers

The Zeus^™^ Robotic Surgical System from Computer Motion consisted of three robotic arms attached to the side of the operation table. In this device, the surgeon used small hand held joysticks to control the movements of the arms. It was used in 1998 for the first robot-assisted surgery in the USA and in 1999, the first robotically-assisted, closed-chest, beating heart cardiac bypass was performed in London using this device. This device was used for tele-surgery in 2001 when a surgeon in New York performed cholecystectomy at a center in France.

In 1995, a group of physicians and engineers founded Intuitive Surgicals Inc. based on their research and training done at the Stanford Research Institute, USA. In 1997, their device, the da Vinci Surgical System, became the first assisting surgical robot based on the telesurgical principle to receive FDA approval for laparoscopic surgery and the first telesurgical cholecystectomy using this device was performed in Brussels. A prototype endowrist device was used in 1998 to perform the first mitral valve repair with the da Vinci. In 2003 Intuitive Surgical Inc. acquired Computer Motion Inc. to become the leader in operative surgical robotics.

### The da Vinci^®^ device[2]

The da Vinci^®^ system is currently the most widely used robotic device in surgery. There are over 220 systems installed worldwide including 12 in Asia and 3 in India. From about 1500 robotic procedures in 2000, over 20,000 robotic surgeries were performed in 2004. One of the largest growths has been in Urology where from 36 radical prostatectomies in 2000, the number has increased to 8000 in 2004. This constitutes over 10% of all radical prostatectomies performed in the USA. The device consists of a surgeon console, patient-side cart, EndoWrist^®^ Instruments, and high resolution 3D Endoscope.

#### Surgeon Console:

The surgeon operates the robot from a console that is placed away from the patient operating table and is connected to the robot with cables. A magnified 3 dimensional image is transmitted to this console from the endoscope similar to the traditional laparoscope placed inside the patient through a port and held by one of the arms of the robot. The console also contains the “masters” and pedals which are moved by the surgeon and which in turn move the instruments in the robot's arms. The console also has the facility to “scale” the movements that help eliminate tremors.

#### Patient-side Cart:

This is the main “robot” with its three or four arms. One of the arms holds the laparoscope providing 3-D images while in the other two or three arms, specialized instruments are placed that enter the body through ports similar to laparoscopy ports. The surgeon at the console controls these instruments and the laparoscope.

#### Detachable Instruments:

The Endowrists^®^ exist at the tips of the detachable laparoscopy instruments in the robotic arms. Unlike traditional laparoscopy instruments where the tip allows very limited movement, these instruments have 7 degrees of freedom, mimicking the human hand. There are various types of instruments for grasping, dissection, cautery, suturing etc. The instruments can be made to rotate in full circles. The surgeon is also able to control the amount of force applied and filter out hand tremors and scale movements. As a result, the surgeon's large hand movements can be translated into smaller ones by the robotic device.

#### 3-D Vision System:

The camera and laparoscope unit of this system uses two independent image sensors that are blended to provided a 3 dimensional vision. This magnified high-resolution real-time image provides over a thousand frames of the instrument position per second and filters each image through a video processor that eliminates background noise.

### Advantages of the da Vinci device

Robotic assistance has certain well-recognized advantages over traditional laparoscopy.

#### 3 Dimensional vision:

The integrated 3 dimensional vision provided by the robotic system allows depth perception that standard 2 dimensional vision of traditional laparoscopy does not. This greatly enhances surgeon dexterity and precision and decreases the number of false movements. The magnified view adds to the confidence and dexterity.

#### Endowrist^®^:

One of the biggest problems of traditional laparoscopy is the limited movements available at the tip of the instruments. Most instruments allow only two or three degrees of freedom. The endowrist, however, allows seven degrees of freedom similar to the human hand. The end result is something akin to inserting the surgeon's hand directly inside the port. The instruments allow circular rotation, something even the human wrist does not allow and makes manipulation extremely simple.

#### Stability:

The da Vinci device allows “scaling” of the surgeon's movements from the master console. This means that the machine is able to convert large movements of the hand into smaller movements of the instruments, thus eliminating tremor and providing stability to the instruments. This allows minute and complex procedures to be performed with greater precision.

#### Ability to perform complex procedures:

A natural consequence of the above two attributes is the ability to perform complex procedures that may be difficult to achieve with pure laparoscopy. This includes reconstructive surgeries such as radical prostatectomy, cystectomy and pyeloplasty where difficult suturing can be made simple by this device.

#### Decreased learning curve:

The ability of the device to mimic human movements minimizes the learning curve. 3 dimensional magnified image adds to this by making the entire procedure similar to open surgery.

#### Laparoscopy naïve surgeons:

The robotic device has allowed laparoscopy naïve individuals to perform complex minimal invasive procedures. Menon et al[3] report that robotic assistance permitted them to graduate from open surgery to laparoscopy without mastering the traditional laparoscopy technique. Their results are as good if not better than traditional laparoscopy.[4] Ahlering and colleagues[5] reported that a surgeon with experience in open prostatectomy but no experience in laparoscopy could perform robotic radical prostatectomy at a skill level equivalent to that of skilled laparoscopic surgeons after more than 100 traditional laparoscopy procedures. This advantage of robotic assistance would be particularly useful in regions such as India where experience of laparoscopic radical prostatectomies is limited.

#### Ergonomics:

One of the least considered issues in laparoscopy is that of surgeon fatigue. Laparoscopy is a difficult surgery often requiring hours of awkward surgeon positioning resulting in significant surgeon fatigue. The robotic device allows the surgeon to sit comfortably at the console and operate. This may not only help increase surgeon output but may also help decrease complications that may occur due to surgeon fatigue.

#### Telementoring and telesurgery:

Telesurgery devices have been used to perform remote telesurgery. This allows surgeons situated miles away from the patient to perform surgery without actual contact. This can potentially have a major role in large countries such as India where expert help is not universally available. However, at least for the time-being, the costs of the device preclude this from being a major reason for its use.

### Disadvantages

The main drawback to this technology is the cost. The device itself costs upwards of INR 5,00,00,000 and then requires annual maintenance and disposable instruments. This has probably been the biggest hindrance to its widespread use but, as with other new technologies, this is likely to come down with time and greater availability.

Another major challenge facing surgeons who train on this device was that they felt hindered by the loss of tactile or haptic sensation. Newer technologies are being developed to overcome this problem and may be resolved within some time.

### Urological applications

While it was initially described for and used in cardiac surgery, the majority or robotic applications today are in the field of Urology. Laparoscopy provides an ideal modality for the management of a number of urologic organs such as the prostate, adrenal and kidney which are deep seated with the body and require incisions much larger than the pathology they are used to treat. A number of these procedures involve extensive reconstruction, a technique difficult to master laparoscopically with a steep learning curve. The robot provides an exquisite solution to these issues allowing relatively less experienced laparoscopists the ability to offer minimally invasive surgery to their patients with a shorter learning curve than with pure laparoscopy with improvement in technical performance.[6,7,8]

The first urological robot, also known as URobot was the PROBOT in 1989 which was used in clinical trials for transurethral resection of the prostate.[9] In 1994, Potamianos looked into a robotic system to assist in intraoperative percutaneous renal access.[10] The access needle was positioned by hand as prescribed by a computer, which formed the calyx location from multiple C-arm X-rays. Later, in vitro experiments that evaluated the system performance showed a targeting accuracy of less than 1.5 mm. The first robot to receive FDA approval was AESOP (Automated Endoscopic System for Optimal Positioning) from Compute Motion Inc. and this device offered 6 degrees of freedom. The Aesop and its variants were used in laparoscopic urology for holding the camera in a steadier and more effective manner than human assitance.[10] AESOP was one of the first to control laparoscopic tools in urologic surgery with its manipulator arms.

The possibility of stereotactic-robotic assistance using an interface was first reported in 1997.[11] The authors successfully punctured the desired calyx in 10 out of 12 procedures using a robotic system. The following year, the same authors described their robotic system “PAKY” which permitted the insertion of a needle in both in-vitro porcine model and actual patients using fluoroscopic guidance.[12] The device was successful in each of its attempts within a mean access time of 8.2 min.

### Radical prostatectomy

Greater awareness and wider availability of screening tests such as PSA have made carcinoma prostate a more common clinical diagnosis. Progressively larger number of patients is being diagnosed in the early organ confined stages of the disease where radical prostatectomy offers a possible cure. This has led to a massive increase in the number of these surgeries being performed every year and in the USA, where screening is widely prevalent, over 75,000 radical prostatectomies are performed every year.

Radical prostatectomy is a difficult procedure and is associated with significant morbidity such as urinary incontinence and erectile dysfunction. There is a natural attempt to minimize this morbidity and also that of the large incision of open surgery and minimal invasive techniques are being developed to achieve these ends.[13] Laparoscopic radical prostatectomy is one of the most difficult laparoscopic surgeries. It has been categorized variously as being difficult to very difficult.[14] Despite there being a large number of centers performing laparoscopic urology, few offer laparoscopic radical prostatectomy as a routine and there are few reports on this procedure.[13,15,16]

In the few years since the initial robotic radical prostatectomies were reported, this procedure has emerged as the single largest indication for the use of the robot.[4,5,17,18] The robot is particularly suited for this surgery because of the small and deep working space, need for precise dissection of apex of the urethra, preservation of the neuro-vascular bundle and reconstruction of the urethra-vesical junction.

Majority of current literature on robotic prostatectomy has come from the Vattikuti Urology Institute at the Henry Ford Hospital, Detroit.[3,4,18–22] Investigators at this institute transitioned to robotic prostatectomy after a period of mentoring in laparoscopic radical prostatectomy. They have prospectively gathered data on their cases since the inception of their robotic prostatectomy program in October 2000. This program is unique in having a dedicated teaching program and began with extensive laparoscopic mentoring during its first year.

Robotic radical prostatectomy is performed most often using the Vattikuti Institute Prostatectomy (VIP) technique that they have described.[4,20,21] The procedure involves 6 ports, three used by the robot and three by two patient-side surgeons. A 12-mm port is placed at the umbilicus for the binocular scope of the robot. Two 8-mm ports are used for the robotic instrument arms and are placed approximately 10 cm from the midline on a line joining the anterosuperior iliac spine to the umbilicus. Two additional ports are placed in the right side for retraction and suction by the first assistant and for the insertion of sutures. The lateral one is a 10-mm and the medial one is a 5-mm port. A 5 mm port is placed in the left flank, slightly inferior to the left robotic port. The robot is then docked to the various ports

An initial incision is made just above the pubic symphysis starting on either side of the medial umblical ligaments and ending with urachal transection. The extraperitoneal space is developed and the bladder is dropped posteriorly. The puboprostatic ligaments are generally left intact and the urethra is dissected to place the dorsal vein stitch. The bladder neck is identified and divided anteriorly to expose the urethral catheter that is then used to retract the prostate caudally. The posterior bladder neck is divided to expose the seminal vesicles which are then dissected off the posterior surface of the bladder. An articulated robotic scissors is used to incise the prostatic fascia anterior and parallel to the neurovascular bundles. The posterior dissection plane is within layers of Denonvillier fascia. Unlike open prostatectomy that shows a single layer, the magnified robotic vision shows multiple layers of this fascia. Dissection is continued in this plane, leaving an added protective fascial layer over the rectum. Precise periurethral biopsies are sent for frozen section before division of the urethra. Urethrovesical anastomosis is performed using two 3-0 braided Monocryl^®^ sutures, one dyed and one undyed. Using the dyed end, the anastomosis is started by passing the needle outside in at the 4-o’clock position on the bladder and inside out on the urethra. Suturing is continued clockwise up to the 10-o’clock position. Suturing is then begun with the undyed end of the suture from outside in on the urethra and then inside out on the bladder. This suture is run counterclockwise up to the 11-o’clock position. The needles are cut off and the free dyed and undyed ends are tied together.

While presenting the results of their large series of over 1100 cases, Menon *et al*^4^ reported an operating time of 70–160 min including 20–40 minutes spent in placing the ports, lysing any adhesions, retrieving the specimen and closing the port sites. The blood loss ranged from 50-150 ml with no blood transfusions. They also discharged over 95% of their patients within 24 hours. Total continence was achieved in 96% patients by 6 months and return of sexual function in 82% of previously potent patients below 60 years of age. There was no mortality and acceptable morbidity. They also found their results in terms of continence and erection to be better than those for open or laparoscopic prostatectomy.

The University of California at Irvine compared the results of 60 open prostatectomy patients with 60 of their most recent robotic cases after excluding the learning curve of the intial 45 robotic procedures.[23] The operating time for robotic surgery was 231 min compared to 214 min for open surgery. The positive surgical margin rate was 16.7% and 20% for the robotic and open surgery respectively. Robotic prostatectomy compared favorably with open surgery in terms of blood loss (103 *vs* 418 cc), transfusion rate (0% *vs* 2%), complication rate (6.7% *vs* 10%) and length of hospital stay (25.9 *vs* 52.8 hours). Urinary continence was 75% in both groups at 3 months.

Cathelineau et al[17] reported the Montsouris experience with robotic prostatectomy in 105 patients. The initial 70 cases were performed through a transperitoneal approach while the more recent 35 using an extraperitoneal approach. Median operating time was 180 minutes and the median blood loss was 500 ml with a transfusion rate of 6%. The overall positive surgical margin rate was 22%.

Robotic radical prostatectomy is clearly emerging as the frontrunner in new technologies in urology and these early results suggests that it may obtain a preeminent place in the management of prostate cancer.

### Renal surgery

The use of the robot for renal surgery is less well defined. Most renal procedures such as a nephrectomy are standardized laparoscopic procedures requiring no reconstruction. However, radical nephrectomy, radical nephroureterectomy and reconstructive renal and ureteral surgery are difficult for naïve laparoscopic surgeons.

In the first description of a robot assisted nephrectomy, Kavoussi et al[24] controlled the camera through a robot, while the surgery was performed laparoscopically by another surgeon. Gill and colleagues[25] described the first completely robotic, telepresent nephrectomy in a porcine model. They were technically successful in all their patients with longer operative times than conventional laparoscopy and they also showed that the da Vinci system was more intuitive and allowed shorter operating times than the Zeus system.[26]

The first telerobotic nephrectomy in a human was performed using a Zeus robotic surgical system with two arms for surgical manipulation and an AESOP robotic arm to control the camera.[27] Subsequently, a series of 18 robot assisted laparoscopic nephrectomies was reported where the operative time with the robot was longer.[28] The telementoring capability of the robot was demonstrated in 2000 when a radical nephrectomy was performed in Singapore by a surgeon in USA.[29]

However, there have been no consistent series of cases reported in the literature on the use of the robot for nephrectomies. One of the principal reasons for this is probably the absence of benefit perceived with purely ablative procedures which have become standardized in pure laparoscopy.

A more likely application for the reconstructive skills of the robot-assisted technique is nephroureterectomy with removal of bladder cuff and partial nephrectomy. Endoscopic resection, pluck removal, stapling and free hand suturing are all used to manage the bladder cuff in a radical nephroureterectomy.[30] Free hand suturing in the depth of the pelvis could be aided through robotic assistance. Similarly, management of the open pelvicalyceal system and bleeding parenchyma following a partial nephrectomy may be simplified using the robot. Pedraza et al[31] reported the use of robotic assistance in a bilateral hemi-nephroureterectomy for duplicated ureters and non-functional upper poles. The robot was used for hilar dissection and isolation of the renal pole while the remaining procedure was completed through pure laparoscopy.

Laparoscopic donor nephrectomy for live-related renal transplantation has become established in over a 100 centers worldwide.[32,33] This technique with its lower morbidity may have contributed to an increase in the number of donor nephrectomies performed, thus helping minimize the gap between donors and recipients.[32] The robot has also found a use in this surgery, considering the immense meticulousness required in its performance. Horgan et al^34^ used the da Vinci robot for 12 donor nephrectomies and all kidneys functioned immediately. In comparison with laparoscopic donor nephrectomy, they noted shorter hospital stay and lower blood loss in the robotic group. They also reported a relatively longer length of vessels retrieved in the robot-assisted group when compared with the pure laparoscopy group. Robot assisted surgery could be especially useful in the harvest of right-sided kidneys where due to a shorter length of renal vein, it sometimes needs to be divided as it enters the vena cava.

Laparoscopic pyeloplasty for uretero-pelvic junction obstruction (UPJO) has become a standardized procedure with success rates equivalent to open pyeloplasty and minimal morbidity due to its less invasive nature.[35] Robotic technology is ideally suited to decrease the technical difficulty in this reconstructive procedure. Sung et al[36], in a porcine study, demonstrated the technical feasibility of robot assisted pyeloplasty. Gettman and colleagues reported their experience of robotic assisted dismembered pyeloplasty in 9 patients with a mean operative time of 138 minutes and no intra-operative complications or conversion.[37] They also reported shorter operative and anastomotic times with the robot when compared with pure laparoscopy.[38] This surgery has highlighted one of the basic advantages of robotic assistance in that even laparoscopy naïve surgeons have directly proceeded to robot assisted laparoscopy.[39,40]

### Adrenalectomy

The deeply situated adrenal gland is ideally suited for a laparoscopic approach. Most tumors are small and require a large incision for open surgical access. After the initial porcine studies at Cleveland Clinic,[21] Horgan et al[41] reported the first human adrenalectomy using the robot in 2001. There have been a number of subsequent reports on the use of robot assistance for adrenalectomy and while there was a higher operative time for robotic surgery, it progressively decreased with increasing experience.[42,43]

### Cystectomy and urinary diversion

Radical cystectomy is the treatment of choice for patients with muscle invasive, carcinoma urinary bladder. Removal of the bladder requires construction of an alternative urinary drainage system. Advances in the use of bowel segments in Urology has lead to the creation of continent urinary diversions and orthotopic neobladders in order to provide the best functional outcome to the patient. Construction of the neobladder requires significant surgical skills. There have been extremely few reports of this being done through pure laparoscopy, primarily because of the extremely long operative times and complexity of the procedure.[44,45,46] The availability of the robot and its dexterity have opened the possibility of an entirely laparoscopic approach to this procedure.

The initial report by Menon et al[47] described the technique of robotic cystectomy. In these patients, the diversion was constructed extra corporeally but the Vesico-enteric anastomosis was performed robotically after re-creation of the pneumoperitoneum. Subsequently, complete laparoscopic procedures using the robot have been described.[48–50] With the possibility of fertility-preserving cystectomy with orthotopic neobladder in females, robotic radical cystectomy may well become the standard of care for this debilitating condition.

### Others

Robots are also being used to help enhance surgical skills and training. Hoznek et al[51] describe the development of a surgical model that can assist the surgeon in choosing the best possible port position for the robot in order to reach the target organ. It also considers data such as optimal tissue handling, ergonomy, visibility and instrument maneuverability. Such models are also being used for surgeon training. They also described a cadaveric renal transplantation with the entire vascular and uretero-vesical anastomosis being performed by a remote surgeon using a robot.[52] The robot has also been used in the performance of a vaso-vasotomy for recanalization of the vas-deferens. The small lumen and precise anastomosis is simpler to perform with the robot and may become feasible for microscopy-naïve surgeons.[53]

Management of vesicoureteric reflux using ureteric reimplantation may also be feasible using robotic assistance. For the extravesical Lich Gregior technique, a three-port approach with incision on the detrusor followed by reapproximation over the in-laid ureter is performed.[54] In an experimental model, Olsen et al[55] described an intravesical repair of bilateral reflux using the Cohen technique of reimplant. The robotic ports are placed into the bladder through the abdomen after saline distension. The bladder is kept distended with gas insufflation while the surgery is completed.

## CONCLUSIONS

Robot assistance is emerging as a significant adjunct to pure laparoscopy. It has to be viewed as a tool for laparoscopy rather than as independent surgical modality. The initial reports cited above attest to its safe applicability in surgeries that are otherwise feasible through pure laparoscopy. The main advantages for this technology are its enhanced dexterity, precision and ergonomics. It has also enabled availability of laparoscopy to patients who would otherwise be candidates for open surgery. The lack of significant experience and training opportunities for pure laparoscopy makes it imperative that easier learning tools such as robotic assistance are available to surgeons.

The issues of cost of equipment and surgery are major hindrances to the widespread use of this technology. It is difficult to expect a robotic revolution if the costs remain what they are today. However, expansion of indications and increasing use will help lowering of equipment costs as more machines are sold.
